# P02.187. Schematic body drawings (mSBD) as an outcome measure for CAM interventions in chronic back and neck pain

**DOI:** 10.1186/1472-6882-12-S1-P243

**Published:** 2012-06-12

**Authors:** M Rasche, R Lauche, R Lüdtke, S von Hein, F Saha, T Rampp, J Langhorst, G Dobos, F Musial

**Affiliations:** 1 Complementarity and Integrative Medicine, University of Duisburg-Essen, Essen, Germany; 2 Karl und Veronica Carstens Foundation, Essen, Germany; 3 NAFKAM, University of Tromsø, Tromsø, Norway

## Purpose

Body image may be distorted in chronic back pain patients (Moseley 2008). We thus asked pain patients to complete (by drawing) pre-defined, printed body schematics, which were lacking the outlines of the particular body parts affected (neck and back). This way the patients were free to complete the outlines according to their subjective perception of their body. The primary aim of this study was to evaluate these drawings as an outcome tool for pain related CAM interventions.

## Methods

Ninety-one patients who participated in two waiting-list controlled randomized trials on wet cupping in chronic neck pain (N=45) and dry cupping in chronic low back pain (N=46) were included. Each patient completed two drawings, one pre and one post intervention. Each drawing was evaluated by three different raters by 37 pre-defined items which aimed to describe its characteristics. Raters were blind to group assignment, intervention, order of drawings, gender, and whether patients benefited from therapy. Inter-rater reliabilities were determined by linearly weighted Cohen‘s kappa (κ) coefficients (Berry and Johnston 2008).

## Results

All kappa coefficients were significant and only two were lower that κ=.40. Raters agreed on group assignment (κ=.57) and on the order of the drawings (κ=.55). The highest level of agreement (κ = .66 to .74) were found for the six items asking for changes in pain related areas. All other values were acceptably high (κ = .40 - .60). Furthermore, consistent correlations between the raters’ judgements for pain related items, in particular regarding the trapezius muscle and the patients’ pain ratings, were found.

## Conclusion

Drawings represent a particularly difficult material to evaluate, nonetheless the inter-rater reliability was fairly good. Evidently, the schematic body drawings conveyed specific information about pain related changes in body scheme and should be regarded as an additional tool for the assessment of treatment effects in pain.

